# Self-Organized Behavior Generation for Musculoskeletal Robots

**DOI:** 10.3389/fnbot.2017.00008

**Published:** 2017-03-16

**Authors:** Ralf Der, Georg Martius

**Affiliations:** ^1^Institute for Computer Science, University of LeipzigLeipzig, Germany; ^2^IST AustriaKlosterneuburg, Austria; ^3^Autonomous Learning Group, Max Planck Institute for Intelligent SystemsTübingen, Germany

**Keywords:** self-organization, robot control, musculoskeletal, tendon-driven, learning, anthropomimetic, self-exploration

## Abstract

With the accelerated development of robot technologies, control becomes one of the central themes of research. In traditional approaches, the controller, by its internal functionality, finds appropriate actions on the basis of specific objectives for the task at hand. While very successful in many applications, self-organized control schemes seem to be favored in large complex systems with unknown dynamics or which are difficult to model. Reasons are the expected scalability, robustness, and resilience of self-organizing systems. The paper presents a self-learning neurocontroller based on extrinsic differential plasticity introduced recently, applying it to an anthropomorphic musculoskeletal robot arm with attached objects of unknown physical dynamics. The central finding of the paper is the following effect: by the mere feedback through the internal dynamics of the object, the robot is learning to relate each of the objects with a very specific sensorimotor pattern. Specifically, an attached pendulum pilots the arm into a circular motion, a half-filled bottle produces axis oriented shaking behavior, a wheel is getting rotated, and wiping patterns emerge automatically in a table-plus-brush setting. By these object-specific dynamical patterns, the robot may be said to recognize the object's identity, or in other words, it discovers dynamical affordances of objects. Furthermore, when including hand coordinates obtained from a camera, a dedicated hand-eye coordination self-organizes spontaneously. These phenomena are discussed from a specific dynamical system perspective. Central is the dedicated working regime at the border to instability with its potentially infinite reservoir of (limit cycle) attractors “waiting” to be excited. Besides converging toward one of these attractors, variate behavior is also arising from a self-induced attractor morphing driven by the learning rule. We claim that experimental investigations with this anthropomorphic, self-learning robot not only generate interesting and potentially useful behaviors, but may also help to better understand what subjective human muscle feelings are, how they can be rooted in sensorimotor patterns, and how these concepts may feed back on robotics.

## 1. Introduction

Control is a ubiquitous theme of life and technology. When reaching for a cup of coffee or walking through the mountains, our nervous system controls all movements with great ease, despite the great uncertainty involved in controlling the muscles, the complexity of the task and many other factors. That this simplicity is an illusion is seen as soon as trying to program a robot for doing a task. While the complexity of programming stands as a challenge for decades, in recent times considerable progress has been achieved by new materials (Kim et al., 2013), powerful actuators (Raibert et al., 2008), the improved theory of control (Siciliano et al., 2009), but in particular by the tremendous increase in computational power that allows modeling and physically realistic simulations of very complex systems to improve planning and control (Mordatch et al., 2012; Erez et al., 2013; Posa et al., 2014) and even allows to simulate large controlled muscular body systems (Yamane and Nakamura, 2011), or find new perspectives for artificial evolution (Bongard, 2015) by exploiting super computer power. Also there are a variety of new control paradigms around, best demonstrated by the amazing locomotion abilities of the Boston dynamics robots, like BigDog, PETMAN and others. These are ingeniously engineered systems for realizing a specific set of tasks with their highly specialized bodies. The DARPA challenge also presents numerous examples of progress but also reveals a realm of failures of these systems even under remote control. Alternatively, the so-called embodied AI recognizes that the body can be very helpful in reducing both design efforts and computational load on the controller. The exploitation of the specific properties of the body, sometimes called *morphological computation* (Paul, 2004; Pfeifer and Gómez, 2009; Hauser et al., 2012) is an active field of research with many impressive results, see Pfeifer and Bongard (2006) and Pfeifer and Scheier (1999), opening new perspectives for both robot control and our understanding of human sensorimotor intelligence (Pfeifer et al., 2012).

The embodied approach seems to be favored in systems with strong physical effects, like soft robotic systems or elastically actuated robots, where the engineering approaches may run into severe difficulties. Though there are a number of interesting results, for instance in employing neural learning to obtain goal-directed behavior, e.g., Manoonpong et al. (2007), Shim and Husbands (2012), Toutounji and Pasemann (2014), and Tetzlaff et al. (2014) using fast synaptic plasticity as in this work, or using simplified spring-models (Park and Kim, 2015), a systematic embodied approach for controlling such systems is not available so far. This is not a surprise, given the aim of exploiting the physical dynamics which is strongly embodiment specific. In this paper we will not aim at a general solution to physics based deliberate control but will investigate the possible role of self-organization (SO) and its general phenomenology in robotics. We will devote this paper to systems with extended embodiment, consisting of a Myorobotics arm connected to a physical subsystem with an internal dynamics of its own. The arm is a muscle-tendon driven (MTD) mechanical system with strong embodiment effects. The controller is a one-layer feedforward neural network which may drive systems into self-organization by a specific learning rule—differential extrinsic plasticity (DEP)—as introduced recently in Der and Martius (2015). It was applied to a number of systems in simulation producing a great variety of behavior. In a slightly modified form, it will face here a new challenge with MTD systems with their strong embodiment effects.

To introduce this paper's topics and claims, imagine that you get an object, a half-filled bottle for that matter, attached to the tip of your forearm such that you can neither know orientation nor identity of the object. When sitting in the dark you probably will start doing something, trying to find out about the object's properties. The idea is, while moving the bottle around, you feel the reaction from the water when hitting the walls of the bottle. Intrigued by this signal and driven by curiosity, you may vary the direction of the shaking motion to end up with shaking parallel to the bottle axis, as the strongest and most coherent force response is coming from there. Without vision or any other external information on the attached object, motor signals are based on the sensor values, i.e., the muscle tensions, modulated by the force responses of the subsystem's internal dynamics. Humans will describe this as feeling the muscles (or the embodiment in general) and generating actions out of this feeling. Generally, behavior is a direct result of the agent-environment coupling, here the dynamical contact between the agent, the arm with its “brain,” and the attached object.

Similarly, with DEP learning, the self-excited motion patterns of the arm are guided, or piloted, by the object's internal dynamics. Specifically, an attached pendulum drives the arm into a circular motion, a half-filled bottle produces axis oriented shaking behavior, a wheel is getting rotated, and wiping patterns emerge automatically in a table-plus-brush setting. This is of interest for the self-organized acquisition of behavioral primitives but there is more: as the emerging patterns are object specific, we may say that the robot was able of identifying the object's identity by just the feedback through the (unknown) internal dynamics of the object. Identifying means that our self-learning system responds with a specific sensorimotor pattern for each object attached to the arm. So, this is a cognitive act closely related to the self-organized discovery of Gibson's object affordances, in particular for dynamical interactions, see below. The observation that DEP learning elicits just these subtle effects unknown so far is the central result of this paper.

Acquired with an anthropomorphic robot (arm), these findings may also provide answers to more general questions in human related cognitive science. Specifically, while the phenomenon of feeling the embodiment (and acting out of this feeling) is easy to grasp from the subjective human perspective, understanding it from the objective scientific perspective becomes very demanding. We claim that our experimental investigation with the self-learning anthropomorphic robot may help to better understand what the subjective human feelings are and how they relate to artificial beings so that this knowledge eventually will help building machines that are in behavior closer to humans.

The paper is organized as follows: In the next section we introduce the DEP learning rule for the controller and give a first discussion of properties, in particular of balancing at the edge of instability which is loosely related to the edge of chaos concept. We present in Section 3 the the experiments with the robot, **Figure 3** for an overview of the experimental settings and Table 1 for a list of videos documenting the various experiments. Throughout the paper, we present different methods for the theoretical analysis based on dynamical system theory. Specifically, we introduce in Section 3.5.1 the eigenvalue spectrum of the linearized dynamical operator, in Section 3.5.2 parametric plots for visualizing the “purity” of a behavior, in Section 3.6 local Lyapunov exponents, and in Section 3.7 Hilbert transforms for analyzing more quantitatively the emerging sensorimotor patterns. Central to the paper is the piloting effect introduced in Section 3.3 which explains how the robot may develop a feeling for the internal dynamics of an object, see also Section 3.6 for its relation to the concept of object affordances. This is followed by Section 4 discussing the findings. Some mathematical details are provided in Section 5 (Supplementary Material).

**Table 1:** **Experiments**.

**Title**	**Description**	**Section**	**Video**
Handshake	Human robot interaction by manually imposing a periodic movement	3.4	Video 1 (Supplementary Material)
Arm with pendulum	Suspending a weight from the tip of the arm: self-excitation of a circular pendulum mode	3.5.1	Video 2 (Supplementary Material)
Pendulum responses	Motors are stopped. Recording spring forces of a swinging suspended weight	3.5.1	Video 3 (Supplementary Material)
Shaking horizontal	Horizontally attached bottle, half filled: Response stronger, shaking horizontally, following the axis of the bottle	3.5.2	Video 4 (Supplementary Material)
Shaking vertical	Vertical attachment, half filled: shaking direction mainly along the (now vertical) axis	3.5.2	Video 5 (Supplementary Material)
How to rotate a wheel	Arm attached frontally to a revolvable bar/wheel.	3.6	Video 6 (Supplementary Material)
Rotating wheel II	Parallel wheel—arm arrangement	3.6	Video 7 (Supplementary Material)
Wiping table	Arm with brush starts to wipe a table	3.7	Video 8 (Supplementary Material)
Wiping table modes	Different wiping patterns from reloaded controllers	3.7	Video 9 (Supplementary Material)
Sensor disruptions	With visual input for hand. Camera is turned during behavior. Fast reorganization	3.8	Video 10 (Supplementary Material)
Hand-eye coordination	Coordination develops, such that arm follows a dummy hand	3.8	Video 11 (Supplementary Material)

*The videos can be watched at http://playfulmachines.com/MyoArm-1*.

## 2. Robot behavior as a self-excited physical mode

The controller we propose is a function that receives at time *t* a vector of sensor values $x_{t}\in {\mathbb{R}}^{n}$ and sends a vector of motor values $y_{t}\in {\mathbb{R}}^{m}$. In the applications, we use a neurocontroller realized by a one-layer feed-forward network as

(1) $$\begin{array}{r} y_{i}=g\left(\kappa_{i}z_{i}\right) \end{array}$$

for neuron *i*, where

(2) $$\begin{array}{r} z_{i}=\overset{n}{\underset{j=1}{\sum }}C_{ij}x_{j} \end{array}$$

is the postsynaptic potential and *C*_*ij*_ is the synaptic connection strength to input *j*. We use tanh-neurons, i.e., the activation function *g*(*z*) = tanh(*z*) to get motor commands between +1 and -1. This is also the reason why we did not include a bias term in Equation (1).

**Figure 1:**
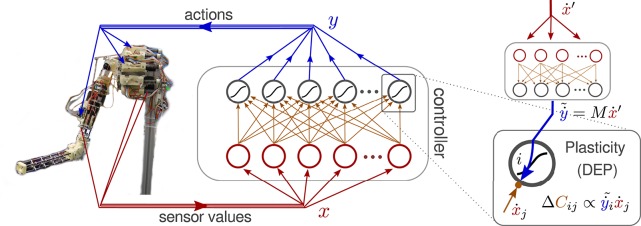
**Neural controller network connected to the Myorobotic arm**. The inset on the right illustrates the synaptic plasticity rule, called differential extrinsic plasticity (DEP) (Der and Martius, 2015). It is driven by a modified differential Hebbian law, multiplying the time derivatives of the incoming sensor values ẋ with the virtual motor values $\tilde{ẏ}$, which are generated by the inverse model (Equation 4) from the next input's derivative ẋ′. In the case of the arm the inverse model is essentially a one-to-one mapping of sensor to motor values.

An important ingredient for the intended self-excitation of behavioral modes is a controlled destabilization of the system. With a fixed *C*, this destabilization is controlled by the gain factors κ_*i*_ in Equation (1) which regulate the feedback strength for each motor channel *i* individually. In the experiments we used the definition^1^ κ_*i*_ = κ/||*C*_*i*_|| where κ regulates the overall feedback strength and ||*C*_*i*_|| is the norm of the synaptic vector of neuron i. The setup is displayed in Figure 1.

### 2.1. Learning dynamics

As we aim at self-organization of behavior, we have to define the control signals in a self-consistent way on the basis of the history of sensor signals alone. Let us introduce $x_{t}^{\prime }=x_{t+\theta }$, the vector of the sensor values received in the next time step, where θ is a time lag with θ = 1 in the derivations given below (time is measured in discrete update-steps, here ^1^/_100_ s).

The self-organized definition of the controller outputs is realized in the following way. Let us postulate the existence of a forward model given by the (possibly state dependent) matrix *A* so that

(3) $$\begin{array}{l} x_{t}^{\prime }=A_{t}y_{t}+\xi_{t} \end{array}$$

where ξ is the modeling error. This describes the physical dynamics over one time step. Introducing *M* which is the inverse or pseudoinverse of *A* we require *y* to be a function of the future sensor values *x*′,

(4) $$y_{t}\overset{!}{=}M_{t}x_{t}^{\prime }$$

Together with the destabilization, Equation (4) displays the essential idea of our approach to make the system active while keeping motor signals compliant with the world dynamics. In a sense, Equation (4) means that the world's responses, represented by *x*′, signals the controller what to do. But of course the world (i.e., the future sensor values $x_{t}^{\prime }$) is also controlled by the controller through the actions *y* (Equation 3). The interplay of these effects is the ultimate reason for the self-excitation of modes by self-amplification of system responses.

However, we cannot use Equation (4) directly for generating the control signal *y* as it contains the future. So, we must find a model for relating the future sensor signals $x_{t}^{\prime }$ to their past, i.e., *x*_*t*_, *x*_*t*−1_, …. In other words, we need a time series predictor for the sensor dynamics. Following the derivation in Section 5.1 (Supplementary Material) we obtain eventually the update rule

(5) $$\begin{array}{l} \tau \Delta C_{t}=M_{t}{ẋ}_{t}^{\prime }{\hat{x}}_{t}^{⊤}-C_{t} \end{array}$$

or in coordinate representation (omitting the time index)

(6) $$\begin{array}{l} \tau \Delta C_{ij}=\underset{k}{\sum }M_{ik}{ẋ}_{k}^{\prime }{\hat{x}}_{j}^{⊤}-C_{ij} \end{array}$$

where $\hat{x}=ẋ||ẋ|{|}^{-2}$, see also Figure 1. The matrix *M* defines the sensor to motor mapping which is one-to-one for normal sensors and negated one-to-one for the delay sensors in the experiments of this paper, see Section 5.2 in Supplementary Material, so the sum in Equation (6) reduces to 2 terms. In general *M* can be more complicated and can be learned in a prior step.

In accordance with earlier work (Der and Martius, 2015), we call this update rule differential extrinsic plasticity (DEP), though there is a difference with ẋ replaced with $\hat{x}$ as the second factor in the update. Equation (5) becomes stationary if

(7) $$\begin{array}{l} C_{ij}=\underset{k}{\sum }M_{ik}\langle {ẋ}_{k}^{\prime }{\hat{x}}_{j}^{⊤}\rangle \end{array}$$

where 〈…〉 is the moving time average. Equation (7) is an important consequence of the update rule, showing that learning converges toward behaviors with a fixed point in correlation space, here a fixed pattern of velocity correlations in sensor space, corresponding to specific attractors in state space. In principle such a fixed correlation pattern corresponds to any behavior like crawling, walking, running, hopping or the like of any amplitude and frequency. If the controller were sufficiently expressive and the sensor to motor mapping appropriate, any (cyclic) mode could potentially be realized by this correlation learning. With the matrix *M* used in this paper, the spectrum of (stable) behaviors is of course restricted but the variety of the observed motion patterns, see below, is still interesting. To enhance self-organization into periodic patterns, we introduce additional sensors which are copies of the primary sensors but are delayed by a fixed time-delay *d*, see Section 5.3 in Supplementary Material for technical details.

For the analysis in terms of dynamic systems theory to be given below, we will need the dynamic operator

(8) $$\begin{array}{l} L=MC \end{array}$$

which describes the mapping from state *x* to *x*′ for the linearized dynamics (Jacobian of linearized system), see Section 5.1 in Supplementary Material for details. The above learning rule differs from the DEP rule introduced in Der and Martius (2015) by the normalization factor ||ẋ||^−2^ introduced with Equation (6) above. In the experiments this leads to a more continuous activity in the behaviors avoiding potential pauses of inactivity. In relation to our earlier work on predictive information maximization (PiMax) (Martius et al., 2013) there are several differences: the DEP rule uses derivatives of the sensors values for learning where PiMax uses the raw ones, PiMax requires to perform a matrix inversion of the noise-correlation matrix which is not needed here, and finally the resulting behaviors obtained from PiMax get high-dimensional (in terms of attractor dimension, see Martius and Olbrich, 2015 for details) whereas the DEP rule yields low-dimensional behaviors as we will see in the analysis below.

### 2.2. Properties

The irreducible conjunction of state and parameter dynamics creates a meta-system—formed by controller, body, and environment—with a rich variety of all kinds of attractors. These can be deliberately switched by manipulative disturbances, creating an attractor meta-dynamics (Gros et al., 2014). This explains why we observe so many different behaviors in the experiments.

#### 2.2.1. Meta-parameters

Furthermore, there are three parameters in this approach κ, τ and *d*, which act as meta-parameters for changing the “character” of the SO process. κ determines roughly the amplitude of behavior. In the experiments, the appropriate value for κ is easily found: when increasing κ gradually, a critical value κ_*c*_ ≈ 1 is eventually reached. Using κ > κ_*c*_ the amplitude *a* of an emerging motion pattern is roughly *a* ∝ κ − κ_*c*_ for small *a*. For larger κ the non-linearities come stronger into play such that the amplitude is never above 1. The time lag of the delay sensors *d* determines the preferred frequency. The parameter τ determines the time scale for taking previous sensor values into account. This has effects on how quickly the controller parameters are wandering around if not yet in a stationary behavior. It is advisable to have it similar or larger to the period of the expected behavior.

#### 2.2.2. Least biasing

The implementation of the controller is explicitly given by Equation (1) together with the update rule Equation (5) which obviously has no system specific components. In the experiments we start always with the least biased initial condition, putting the controller matrix *C* = 0 so that all actuators are in their central position. A basic requirement for a “genuine” approach to SO is its independence of specific properties of the controlled system. Obviously, this is realized here in an ideal manner by both the structure of the approach and because there is no specific goal, no target signal, no platform specific information and no biasing.

#### 2.2.3. Theoretical analysis

It would be interesting and helpful if the wide spectrum of self-organizing behavior could be given a quantitative analysis. In goal oriented learning this can be done by some performance criterion, assessing the difference between actual and intended behavior. However, this seems not appropriate in a true self-organization scenario like that of the present paper. Still one may ask for a profound theoretical analysis of what these systems actually are doing. This paper contributes to that task by presenting several such measures which are partly a bit unorthodox but were quite successful for analyzing behavior generated by the DEP learning rule. Central is the use of dynamical systems theory in several aspects. Specifically, we investigate below the eigenvalue spectrum of the linearized dynamical operator *L* = *MC* as introduced in Equation (8), using it for assessing the nature, and the stability of periodic motions, the prevalent modes in this paper. We use local Lyapunov exponents as a more quantitative concept of dynamical system theory, arguing that they may be a first guess for the claimed realization of an edge of chaos system, see Section 3.6 below. Also, parametric plots have proven a viable tool for visualizing the nature of behavior and last but not least, Hilbert transforms of the sensor signals were used for analyzing the phase relations between sensor and motor signals, thereby quantifying the closure of the sensorimotor loop, see Section 3.7.

The nature of the dynamical system generated by the learning rule may also be quantified by a number of methods from complexity theory, information theory (Bialek et al., 2001) and more evolved tools from non-linear dynamics (Kantz and Schreiber, 2004). Akin to this paper are methods for analyzing emergent behavior (Lungarella and Sporns, 2006; Ay et al., 2008; Wang et al., 2012; Schmidt et al., 2013) using information theory. A new quantification based on excess entropy (predictive information) and attractor dimension was recently proposed in Martius and Olbrich (2015) and applied to similar self-organizing behavior as found in this paper. However, there long traces of repetitive behavior where recorded in simulations to estimate entropies. Unfortunately it is impossible to perform this analysis for the fast online learning of the synaptic dynamics, given the time scale of a few seconds or minutes for the behavior generation.

There is some pioneering work in using dynamical systems theory for analyzing behavior generation by fast synaptic plasticity. In Sándor et al. (2015) and Gros (2015), the interesting concept of an attractor metadynamics was introduced which is close to the scenario of this paper. However, their analysis, while pointing in the right direction, is restricted so far to rather simple physical systems in simulation, so that we did not apply it in this paper. Related ideas may also be found in Toutounji and Pasemann (2014, 2016).

#### 2.2.4. Edge of chaos—the working regime for self-organization

An essential feature of our approach is the possibility to chose, by the parameter κ, the working regime at the boundary between stable and unstable dynamics. This working regime may be associated with the somewhat vague “edge of chaos” concept (Langton, 1990; Mitchell et al., 1993; Kauffman, 1995; Bertschinger and Natschläger, 2004; Natschläger et al., 2005). As is known from dynamical system theory, this region is not well defined but is otherwise of eminent interest for understanding both life and creativity in natural and artificial beings. Unfortunately, with systems of the physical complexity considered here, a strict mathematical analysis of this region, e.g., by global Lyapunov coefficients, is out of reach of this paper. Nevertheless, in a sense, one can observe in the videos the edge of chaos hypothesis, i.e., to live somewhere between order and fully developed chaos. In fact, on the one hand the systems react very sensitively on weak perturbations, in particular one may observe that the further development of behavior is determined by the initial kick the system experiences or by the interaction with attached objects with an internal dynamics. This extremely sensitive reaction to perturbations is a signature of chaos. On the other hand, see the pendulum video or the bottle shaking experiments, the system also has a high degree of organization as demonstrated by the emergence of long-lived regular orbits. This is the order aspect of the scenario.

Developing quantitative measures for the edge of chaos regime may get the robotic community interested in this very rich, intellectually appealing, and potentially highly useful branch of dynamical system theory based robotics. But this is a topic of future research.

#### 2.2.5. Platforms for embodied AI

Finally, let us discuss on which platforms our controller is likely to create useful behavior. First of all, the system has to provide sensory feedback about acting physical forces to make embodiment effects perceivable by the controller. This is, for instance, not the case if all perturbations are perfectly compensated by a low-level PID controller. Secondly, there should be sensors reporting a similar quantity as used to control the actuators, e.g., position sensor for position control or force sensors for force control. Additional sensors are typically integrated into the loop if they show a definite response (correlation) to the motor patterns. Thirdly, the behaviors of interest should be oscillatory. Since we only need the main sensor-to-motor wiring information about the particular robot (which can also be learned) and do not require any other specific information, we expect our system to work with a wide variety of machines including soft robots, but this remains for future research.

## 3. Experiments

**Figure 2:**
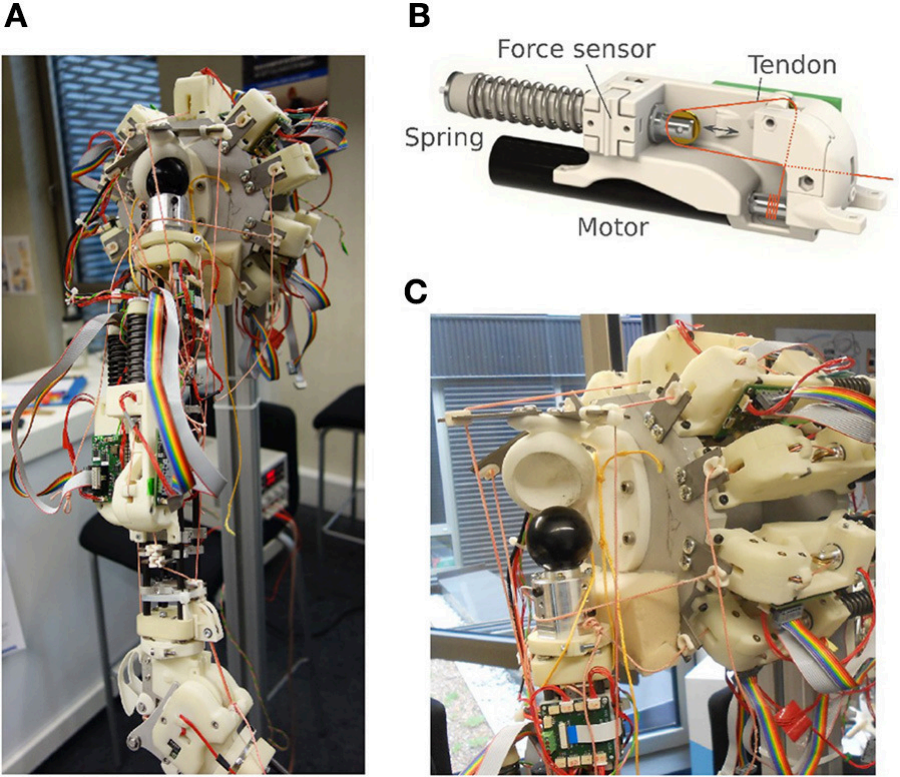
**Myorobotic arm (A)**, a single muscle element **(B)**, and a dislocated shoulder **(C)**. The dislocation happens wickedly as soon as the tendons are getting slack.

The above defined controller was used in the experiments with a tendon driven arm-shoulder system from the Myorobotics toolkit (Marques et al., 2013), see Figure 2. The system has 11 artificial muscles, 8 in the shoulder and 2 in the elbow and one affecting both. However, two of the shoulder muscles where disconnected. The muscles are composed of a motor winding up a tendon connected to a spring, see Figure 2B. The length of a tendon *l* is given by the motor encoders and the spring compression by *f* which is in the interval [−α, 1 − α] where α defines pretension (here α = 0.1). The length of the tendons is normalized to *l* ∈ [−1, 1]. We define the sensor values as

(9) $$\begin{array}{l} x_{i}=l_{i}+\beta f_{i} \end{array}$$

where β regulates the integration of the spring-compression. In the experiments, β was simply set to 1 without further tuning. It is expected that this choice is not critical. After the initialization, where the arm is put in a defined initial position, all tendons are tightened to their pretension, and all *l*_*i*_ are set to zero, the system is put into a position control mode where the controller output *y*_*i*_ defines a target tendon length for each tendon. In the experiments we used the following parameter settings: κ = 0.5, τ = 1*s* (Equations 1, 5), delay sensor lag: 0.5 s (Section 5.3 in Supplementary Material), a time distance between *x* and *x*′ of 0.08*s*, *r* = 10^−3^ (Equation 22), and an update frequency of the control loop of 100 Hz.

### 3.1. Peculiarities of muscle-tendon driven systems

There are a number of features which make the muscle-tendon driven (MTD) systems different from classical robots with joints under rigorous motor control, i.e., the motor positions directly translate into joint angles and into poses. Naively one could think that control is very easy, realized by just pulling the right strings (tendons) for getting a desired arm pose. However, life is much more difficult due to a number of annoying effects. The most obvious effect is seen when tendons are getting slack so that contact with the physical state of the arm is lost altogether. This has to be avoided by keeping a permanent tension on the tendons, which poses another problem: The tension can only be achieved by tightening each tendon up against all the others, each individual tension being reported by the spring length. This means that (i) there are infinitely many combinations of tension forces for a single arm pose and (ii) that the action of a single motor will be reflected in a change of spring length of all other muscles. In other words, actuating a single muscle is reflected by a pattern of sensory stimulation—a whole-body answer.

Furthermore, the combination of friction effects and muscle-pose ambiguity leads to a hysteresis effect. After driving the arm by a sequence of motor commands from pose A to pose B one ends up in a different pose and muscle configuration than *A* after moving back by reversing the motor commands. In general, this makes the translation of a kinematic trajectory for the arm into motor programs difficult, even more so if there are loads and high velocities involved. Also, the classical approach of learning a model by motor babbling becomes problematic because actions cannot be chosen independently.

We conducted several experiments listed in Table 1 which demonstrate the essential features of the control scheme. All experiments are done with the same controller with the same initialization (*C* = 0) so that it is only the physical situation that differs between the experiments.

We strongly recommend consulting the videos for better understanding which can be found at http://playfulmachines.com/MyoArm-1.

### 3.2. Self-regulated working regime

Before presenting the experiments in more detail, let us take a look at the sensorimotor coupling that is created by our controller. One of the crucial features is the self-regulation into a working regime where the tendons are kept under tension even in very rapid motions with notable loads. This is very important as it guarantees the signals from the controller to be executed in a definite way. As a result, in all experiments we never had to face a shoulder dislocation, see Figure 2C, which may happen promptly if tendons are getting loose. This is of some importance as this sensible working regime emerges without any additional tuning or calibrating (Wittmeier et al., 2012) the system. For that, the specific sensor configuration (Equation 9) seems to be important, but we did not study it systematically yet and expect other configurations to work as well. A more rigorous analysis in terms of the local Lyapunov exponents will be give in Section 3.6 below.

### 3.3. The piloting effect. feeling the embodiment

In the Introduction, we presented a thought experiment illustrating the main features of this work. We did not yet carry out this experiment with humans, but the scenario of getting piloted by the subsystem toward activities of strongest response is just what we observe with the learning arm for a series of very different objects, ranging from the pendulum to the wheel to the wiping a table setting. In any of those situations we could not only observe the piloting effect but also support it by quantitative analysis. Let us remember that any motion of the arm impacts on the inner dynamics which reacts back on the arm via the force response of the internal dynamics, like the water hitting the wall of the bottle. These force responses modulate the sensor values (measuring the length of the tendons) and may become self-amplifying under the learning rule as substantiated by the following arguments (which still need more theoretical support). Point one is that these signals, though tiny, generically may be systematic, building correlations over space and time. Examples are the slow swaying motion of the pendulum or the inertia motions of the water. As the DEP rule enhances correlations by the learning process, any systematic signal persisting over the time scale of learning contributes to the correlation pattern with an enhanced strength. In the experiments, the time scale set by τ was one second, about the same as the internal dynamics of the subsystems. This seems to be the main cause of the piloting effect. Furthermore, the learning system was seen to be the host without preferences of a wide spectrum of attractors giving rise to a kind of attractor morphing. Meaning the learning rule changes the dynamics such that the attractors continuously change, all modulated by the systematic force responses from the subsystem. In other words, the learning system has no resistance to being piloted into a resonance with the subsystem. The piloting by the subsystem is the leading mechanism in the experiments described in the following.

### 3.4. Manipulability

The dominance of the physical responses makes the system manipulable as any externally applied forces—like a physical robot human interaction—leave their footprint in the sensor values via the changing spring tension. For instance, the arm can always be stopped by simply holding it. The reason is not that the motors are too weak. Instead, ẋ = 0 is a fixed point of the dynamics of the meta-system to which it relaxes if the mechanical degrees of freedom are frozen manually^2^.

**Figure 3:**
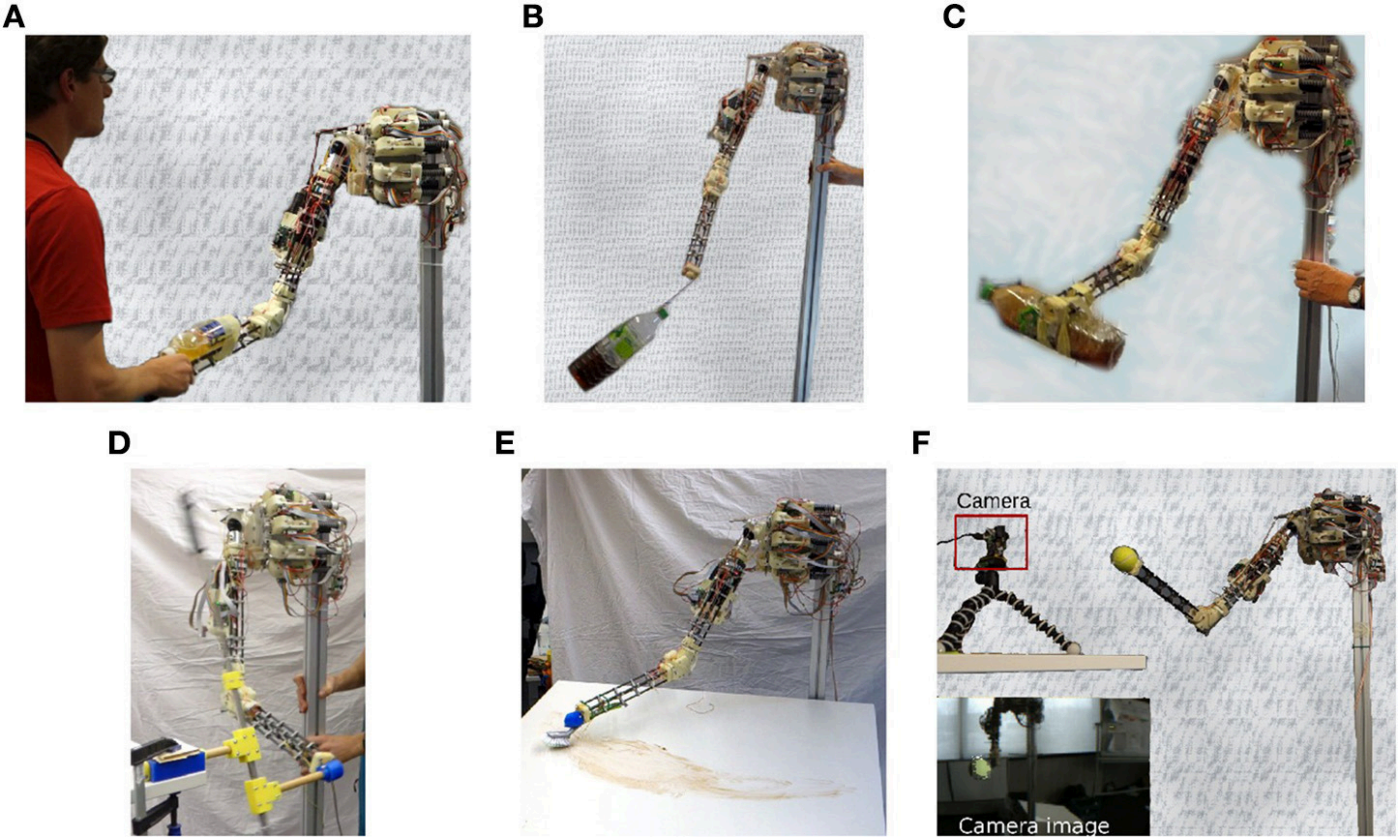
**Experimental setups**. Handshaking **(A)**, pendulum swinging **(B)**, bottle shaking **(C)**, rotating a wheel **(D)**, wiping a table **(E)**, and hand-eye coordination **(F)**. All experiments are performed with the same controller.

**Figure 4:**
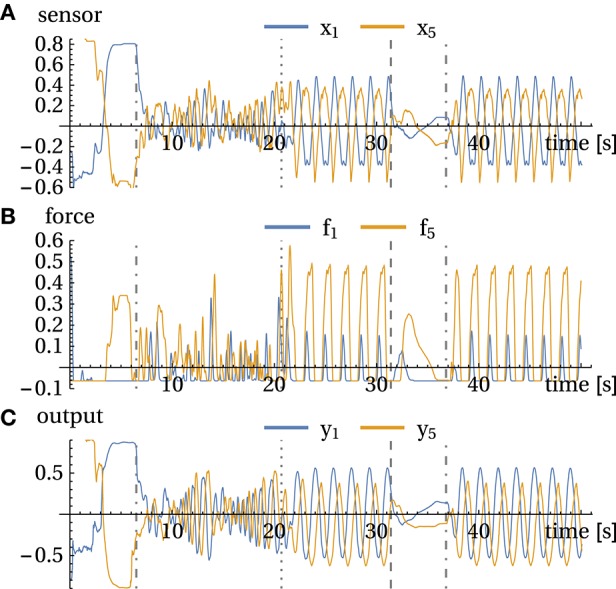
**Handshake experiment**. **(A)** sensor values *x*, **(B)** forces *f*, and **(C)** motor values *y* for channels 1 and 5. Events: 6 s: operator is grasping the arm and starts the handshake; 21 s: freezing of parameters and release at 31 s. 35.5 s: bringing arm into resting position, it stays there until 37 s where it got perturbed. See also corresponding Video 1 in Supplementary Material.

Moreover, the system can be entrained by manual interaction into specific behaviors. We demonstrate this in the handshake experiment, see Figure 3A and Video 1 in Supplementary Material, where the user is trying to move the arm in a periodic pattern. Besides the possibility to train a robot in this way, the most interesting point is the subjective feeling that comes about when interacting with the robot. In the beginning of such an interplay, the robot seems to have a will of its own as it resists the motions the user is trying to impose. But after a short time the robot follows the human more and more and eventually is able (and “willing”) to uphold the imposed motion by itself, see Figure 4. Otherwise, depending also on the human partner, the meta-system of robot and human may “negotiate” a joint motion pattern which might be left if the human quits the loop. This can be understood by realizing that any periodic patterns creates a fixed correlation pattern in Equation (7). If the imposed patterns match one of the stable ones, the robot is controlling this pattern by itself. In fact, in the experiments, one can well observe that a “compliant” human is intrigued to follow the system as much as its own intentions, ending up in an orchestrated human-machine dynamical pattern.

Training of a robot by directly imposing motions is not new. The common approaches generate a kinematic trajectory which is afterwards translated into the motor commands by well known engineering methods. This method may run into some difficulties due to the peculiarities of our MTD system discussed in Section 3.1. With DEP learning, imposing the patterns is a process of creative interaction with the system, see also the training of wiping patterns in Section 3.7.

### 3.5. Emerging modes

As already mentioned above, DEP learning as formulated in Equation (1) drives systems toward attractors in state space corresponding to fixed velocity correlation patterns in sensor space. The selection of a specific attractor may be realized by the self-amplification of a dynamical seed, generically provided by an initial perturbation from e.g., gravitational forces or by tipping the arm.

#### 3.5.1. Self-excited pendulum modes

**Figure 5:**
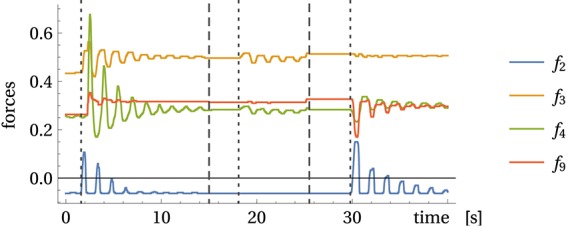
**Force measurement with static arm**. Displayed are the force measurements with swinging bottle but without active arm movements for muscles 2, 3, 4, and 9 (for clarity). Dotted lines indicate when the bottle was manually set into motion and at dashed lines it was stopped, see Video 3 in Supplementary Material.

In a first experiment, we suspend a weight (the bottle) from the tip of the arm, see Figure 3B. With the pivot point (arm) at rest the pendulum may realize ellipsoidal or circular motion patterns with fixed frequency. In general, when considering a pendulum with moving pivot it can perform chaotic motions under certain trajectories of the pivot point. With the pendulum attached to the MyoArm, the motions of the weight exert small inertia forces on the arm which change the spring tensions and thereby leave a footprint in the sensor values. To illustrate this point, Figure 5 displays the sensor reading for the swinging pendulum with the motors being stopped. While being tiny, these reactions are systematic, leading to the self-excitation of resonant modes according to the piloting effect described in Section 3.3 above.

**Figure 6:**
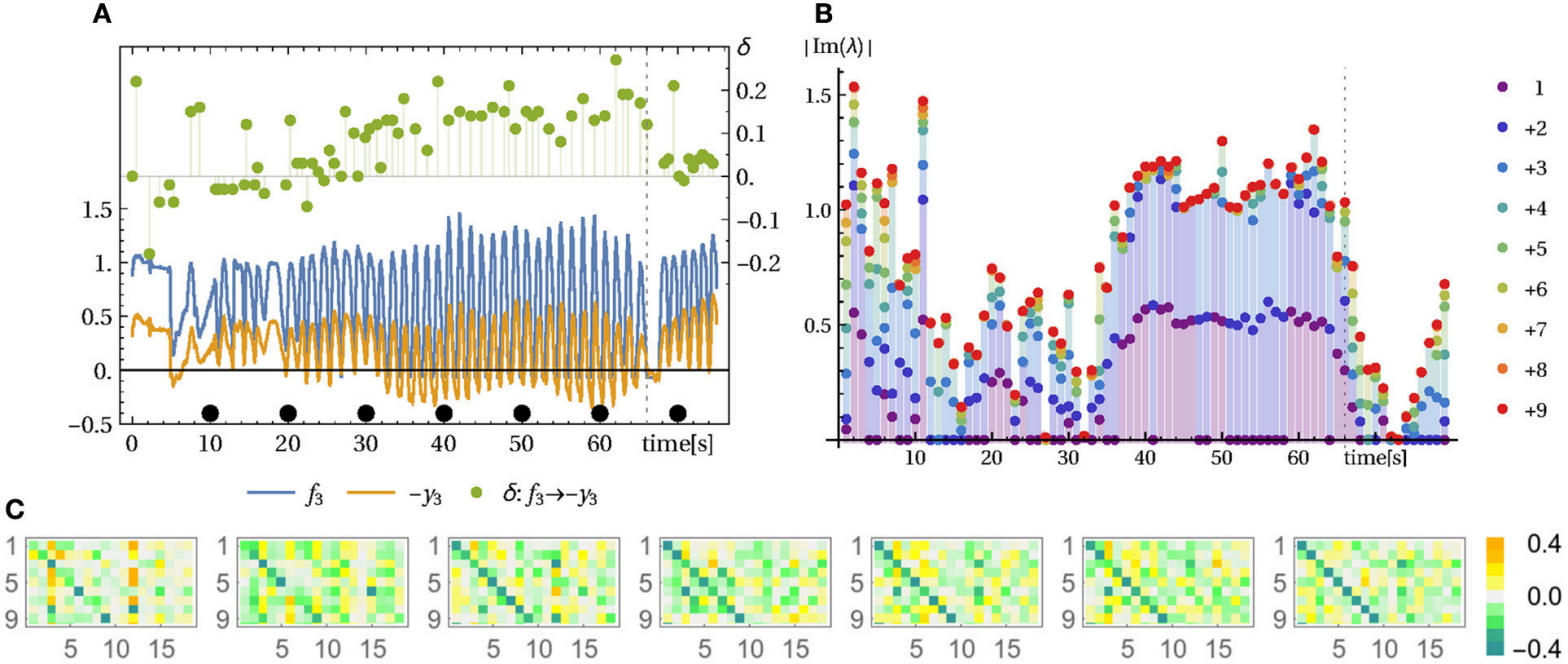
**Pendulum modes**. See Video 2 in Supplementary Material. **(A)** Force sensors and control signal of muscle 3 and their time lag. The measured force (spring compression) and the control signal *y* (desired tendon length) follow a similar trajectory with inverted sign (note −*y*). The time lag δ (right axis in seconds) between force and motor value (same result for other muscles) indicates that initially the control and the environmental influences are not in sync whereas in the swinging mode (from 33 s on) a stable phase/time-lag relation is observed. **(B)** Displayed are the absolute imaginary parts of the eigenvalues of the linearized system dynamics (Jacobian *L*, below Equation 5) (averaged over 1 s) and cumulatively plotted (1, 1 + 2, 1 + 2 + 3, …). During the pronounced oscillation between 35 and 68 s there is one pair of dominant complex eigenvalues. **(C)** Corresponding controller parameter *C* at the seconds 10, 20, …, 70 (from left to right) as indicated by the black dots in **(A)**. At second 66 the string of the bottle was shortened causing the mode to break down immediately, see Video 2 in Supplementary Material.

In Video 2 (Supplementary Material) it can be seen^3^ directly how latent velocity correlations are being amplified to end up in stable circular motion patterns of the pendulum. The experiment starts in a situation where the motor activities have settled to rest, interrupted by occasional bursts leaving irregular footprints in the sensor values. As to the piloting effect, we have to verify that, starting with this irregular behavior, the compound system is driven into a resonance with the pendulum and that this resonance behavior is dominated by the (tiny) force responses of the pendulum. This may be supported by analyzing the time lag between measured force and driving signal (motor commands). As shown by Figure 6A, the incipiently rather irregular phase relation is followed by a constant phase from time *t* > 40 on. This convergence to a stable mode is also seen by the time evolution of the controller matrix *C*, see Figure 6C.

Let us consider here, as a further bit of analysis, the eigenvalue spectrum of the dynamical operator *L* = *MC*, which has proven very useful in this work. Actually, if the system would obey the linearized dynamics, any cyclic behavior should be reflected by the existence of a pair of complex eigenvalues. There might be more of such pairs if there are different frequencies involved. Though questionable due to nonlinearities and deficiencies of the linear operator, this analysis may yield reliable results as seen in the pendulum case: Figure 6B clearly displays just such a pair of eigenvalues with absolute value (not shown) a little above one. All other eigenvalues have a absolute value significantly smaller than one which makes the corresponding modes short lived^4^. The latter point was investigated in terms of the local Lyapunov exponents, see Section 3.6 below, for remarks on that method. Apart from identifying the oscillatory modes, this eigenvalue analysis also confirms the substantial dimensionality reduction which is also known as a signature of self-organization.

#### 3.5.2. Bottle shaking modes

In a next series of experiments we attached a bottle filled with some liquid to the tip of the arm in either horizontal or vertical orientation, see Figure 3C. These experiments are meant to support our hypothesis on the piloting effect, i.e., that, under the DEP learning rule, the emerging motion patterns are defined eventually by force responses of the subsystem. With the bottle, the force response is solely generated by the internal motions of the water, i.e., when the water is hitting either the walls or top and bottom of the bottle. Similar to the pendulum, starting with spontaneous movements, the arm soon reaches an oscillatory mode with strong force answers. In the experiment, the emerging shaking motions are indeed more or less aligned with the axis orientation of the bottle, see Videos 4, 5 in Supplementary Material, in correspondence to the piloting effect.

**Figure 7:**
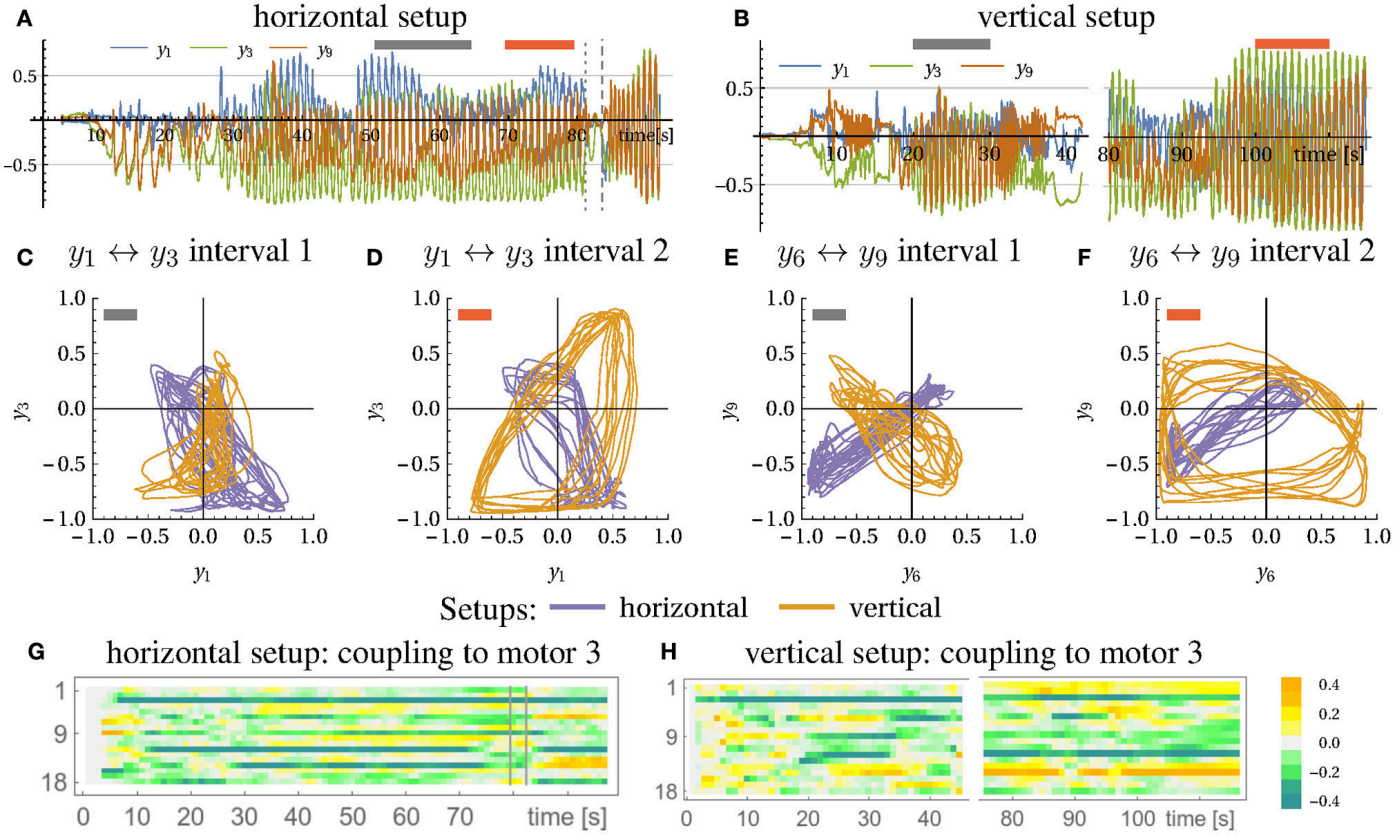
**Horizontal and vertical bottle shaking experiment**. Depicted are the time traces of the motor values for the horizontal setup **(A)**, see Video 4 in Supplementary Material, and the vertical setup **(B)**, see Video 5 in Supplementary Material. At the marked regions (gray and red bar) both setups are compared in **(C–F)** with respect to their motor relation (motor 1 vs. 3 and 6 vs. 9). It is visible that the motions in both setups are mostly orthogonal to each other. **(G,H)** shows the evolution of the coupling of the 18 sensors to muscle 3 over time (corresponding to row 3 in *C*). In both cases the system starts at *C* = 0. In the horizontal case the arm was stopped and released at times indicated by vertical lines.

We also performed a more quantitative analysis by using parametric plots to characterize the state dynamics. Oriented at the arm's geometry, we identified two pairs of motor values (*y*_1_, *y*_3_) and (*y*_6_, *y*_9_) which are expected to be discriminating the direction of the arm movement, i.e., to have different phase relations for the horizontal and vertical arm movements, respectively. When plotting the time course of (*y*_1_, *y*_3_) and (*y*_6_, *y*_9_) in the plane, fixed phase relations translate into typical ellipsoidal figures. In Figures 7C–F we compare the phase relation for the horizontal and vertical setup (violet and orange line, respectively) for two behavioral modes (see Figures 7A,B for the time course and intervals) and indeed find that they are different and often orthogonal to each other. The emerging motion pattern is determined by the axis direction of the bottle, with the reactive forces of the water as the only information for that direction. Metaphorically, the robot can “read” the information about the nature of the environment by just getting into dynamical contact with the latter in a completely self-organized way.

In Figures 7G,H we present the time evolution of the matrix elements *C*_3*j*_ representing the connection to the motor unit 3. As starting from the zero-initialization, one can see how first correlations build up due to the dynamics of the *C* matrix (Equation 5). The following behavior is highly transient until convergence is (roughly) reached where the dynamics gets more stationary. Any perturbation or change in conditions leads to an adjustment of the controller, always aiming for a mode where high velocity correlations appear.

### 3.6. Rotating a wheel

A further example for the piloting mechanism (Section 3.3) and the discovery of dynamic object affordances (as discussed below) is the robot arm connected to a wheel, see Figure 3D. In Der and Martius (2015), the emergence of rotational modes was demonstrated for a humanoid robot with revolution joints and in simulation. With the MyoArm, we have a much more challenging situation. In the experiments, the tip of the arm is attached to the crank of a wheel, implemented as a revolvable bar with weights for giving it the necessary moment of inertia. In Video 6 (Supplementary Material), initially the connection between the arm and the wheel was rather loose so that for small movements there is no definite response from the rotation of the wheel. After improving this connection, an initial push by the experimenter was sufficient to excite a rotation mode that persists over time and is stable under mild perturbations. It is as if the controller “understood” how to rotate the wheel, although it is just the result of force exchange in combination with correlation learning, i.e., by the mechanism described in Section 3.3. When positioning the wheel in parallel to the arm, the modes were emerging even more readily as seen in Video 7 (Supplementary Material). Furthermore, the system may be changed in frequency by changing just the time-delay *d* as shown earlier (Martius et al., 2016).

**Figure 8:**
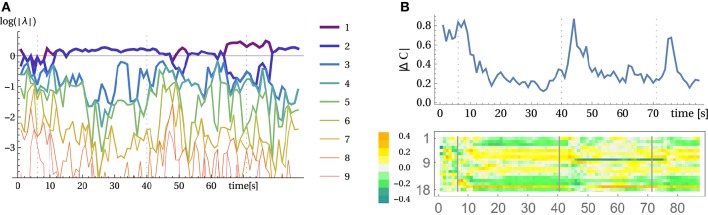
**Analyzing the wheel rotation for the parallel setup**. **(A)** Local Lyapunov exponents of the linearized dynamics, i.e., logarithm of absolutes of the eigenvalues of *L* (see below Equation 5). **(B)** Temporal evolution of the controller matrix *C*. Absolute changes of *C* over one second (top) and changes in the coupling of all sensors to motor 6 (row 6 of C over time). The arm started to rotate at second 7 and at second 40 and 71 the rotation was manually inverted (vertical lines) see Video 7.

For an analysis, we may use here the method of local Lyapunov exponents, given by the eigenvalues of the dynamical operator *L* = *MC* transforming sensor states *x* to *x*′ under the linearized dynamics. Figure 8A displays the results. The point of interest are the two largest exponents which are slightly above zero. They represent the rotational mode. Being above zero means that they are actually instable which was to be expected given the slight destabilization of the system controlled by the parameter κ. However, the system dynamics is kept from exploding by the nonlinearities so that the rotation modes are stable but all other modes have to die out, i.e., their Lyapunov exponents have to be below zero. It is also illustrative to consider the absolute change of the controller matrix as displayed in Figure 8B (top). At the beginning of a new mode the changes are large and then settle to a background level. When, for instance, the rotation is externally changed (second 40 and 71) then again a high rate of change is observed. The coupling of the sensors to motors also changes qualitatively between the modes as illustrated at the example of motor 6 in Figure 8B (bottom).

The constitutive role of the body-environment coupling is also seen if a torque is applied to the axis of the wheel. Through this external force we may give the robot a hint of what to do. When in the fluctuating phase, the torque immediately starts the rotation which is then taken over by the controller. Otherwise, we can also “advise” the robot to rotate the wheel in the opposite direction. This can be considered as a kinesthetic training procedure, helping the robot in finding and realizing its task through direct mechanical influences.

Finally, these results can also be of interest for elucidating the spontaneous discovery of object affordances. Following Gibson (1977) theory of affordances, object affordances are defined as a relation between an agent and its environment through its motor and sensing capabilities (e.g., graspable, movable, or eatable and so on). In this sense, in the same way as a chair affords sitting or a knob affords twisting, the wheel in our experiment affords rotating it, the bottle affords shaking and pouring and so on. This is of immediate interest for embodied AI as affordances are prerequisites for planning complex actions. Because our controller generates dynamic and typically oscillatory movements it can only discover dynamic afforcances, such as shaking, turning etc. but will not find static ones such as sitting on a chair or leaning against a wall.

### 3.7. Wiping

In the case of the wheel setup, above, the embodiment strongly constrains the possible motion patterns. In the next setup the agent-environment coupling imposes a much milder restriction on the behavior: the robot is equipped with a brush and a table is placed in its work-space, see Figure 3E. The table height is about 5 cm above the initialized resting position. Video 8 in Supplementary Material demonstrates how, by the combination of the restricting table surface and the manual force, the robot is guided into the two-dimensional wiping mode. Actually, even without this guidance the system typically learns a wiping behavior, because movements perpendicular to the table are strongly damped such that the directions along the table plane may create the highest velocity correlation and thus dominate the generated motion patterns. Later in this video, the robot is forced by hand into a different behavior.

The analysis of the dynamics during this experiment revealed that the wiping patterns where not stationary as it appeared in the video, but are actually slowly drifting. We devised a method to quantify such high-dimensional oscillatory behavior. It considers the phase difference between the different degrees of freedom. For each oscillatory signal we can associate a phase variable that continuously runs from −π to π using the Hilbert transform. Now we can compute the phase difference between the signals from different sensors, for instance. Post-processing is applied to avoid unnecessary 2π phase jumps and to smoothen the signal for better visibility.

**Figure 9:**
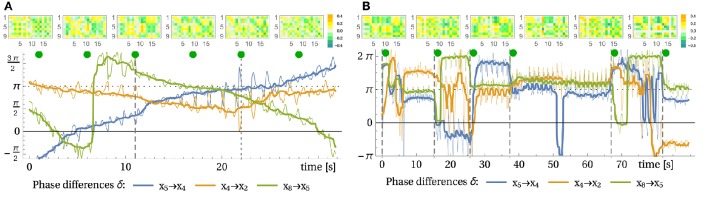
**Learning to wipe a table with a brush and recall of different wiping patterns. (A)** Shown are the phase differences between a selection of sensor values (bottom) and the controller matrices (top) at different points in time indicated by green dots. The thick lines show the sliding median of 2 s windows for better visibility. Note that jumps of 2π are equivalent to no phase change. See corresponding Video 8 in Supplementary Material (Time 0 in the plot is at the first cut in the video). From second 11 (dashed line) to 22 (dotted line) the arm was trained to perform a different movement, which persists for a few seconds until the system drifts away. **(B)** Recall of previously stored behaviors. At vertical dashed lines, a static controller was loaded. Phase differences between a selection of sensor values (bottom) and the controller matrices (top) (times, see green dots). See corresponding Video 9 in Supplementary Material. Observe the transients between the behaviors, which are sometimes long, e.g., 15 s for controller 4.

In a stable oscillation, the phase difference should stay constant over time. In Figure 9A, these phase differences are presented for the wiping experiment. One can see that already before manual interaction, the meta-system is in a transient behavior, with changing phase relations slowly over time. We interpret this as a wandering through the metastable cyclic attractors induced by the learning dynamics. We may also call this a self-induced attractor morphing. During interaction (second 11 onward) the changes are initially stronger, fading out later. After releasing the arm (second 22), behavior persists for a few seconds and then is again drifting away. The corresponding controller matrices also show a significantly different structure in the course of the experiment. With the phase analysis using Hilbert transform we can thus analyze pseudo-stationary high-dimensional motion patterns and we believe this methods is also helpful to analyze other systems where attractor morphing occurs.

So, what appeared as stationary actually was a transient behavior. As explained above, there is a potentially infinite reservoir of attractors in *C*-space, with the learning dynamics slowly and continuously morphing these attractors. Being more or less a speculation so far, this opens a view into a fascinating species of dynamical systems generated by the learning rule in specific agent-environment couplings. Moreover, this also should substantially improve our understanding of the edge of chaos hypothesis as an overarching concept.

Otherwise, by simply storing the weights (*C*) of the controller, these patterns can be collected into a repertoire. Video 9 in Supplementary Material shows the recall of and switching between such wiping modes, see Figure 9B. For the transition into a different mode the controller was changed abruptly, nevertheless a smooth transition into the new behavior occurs, suggesting that most static controllers have a large basin of attraction.

### 3.8. Hand-eye coordination

**Figure 10:**
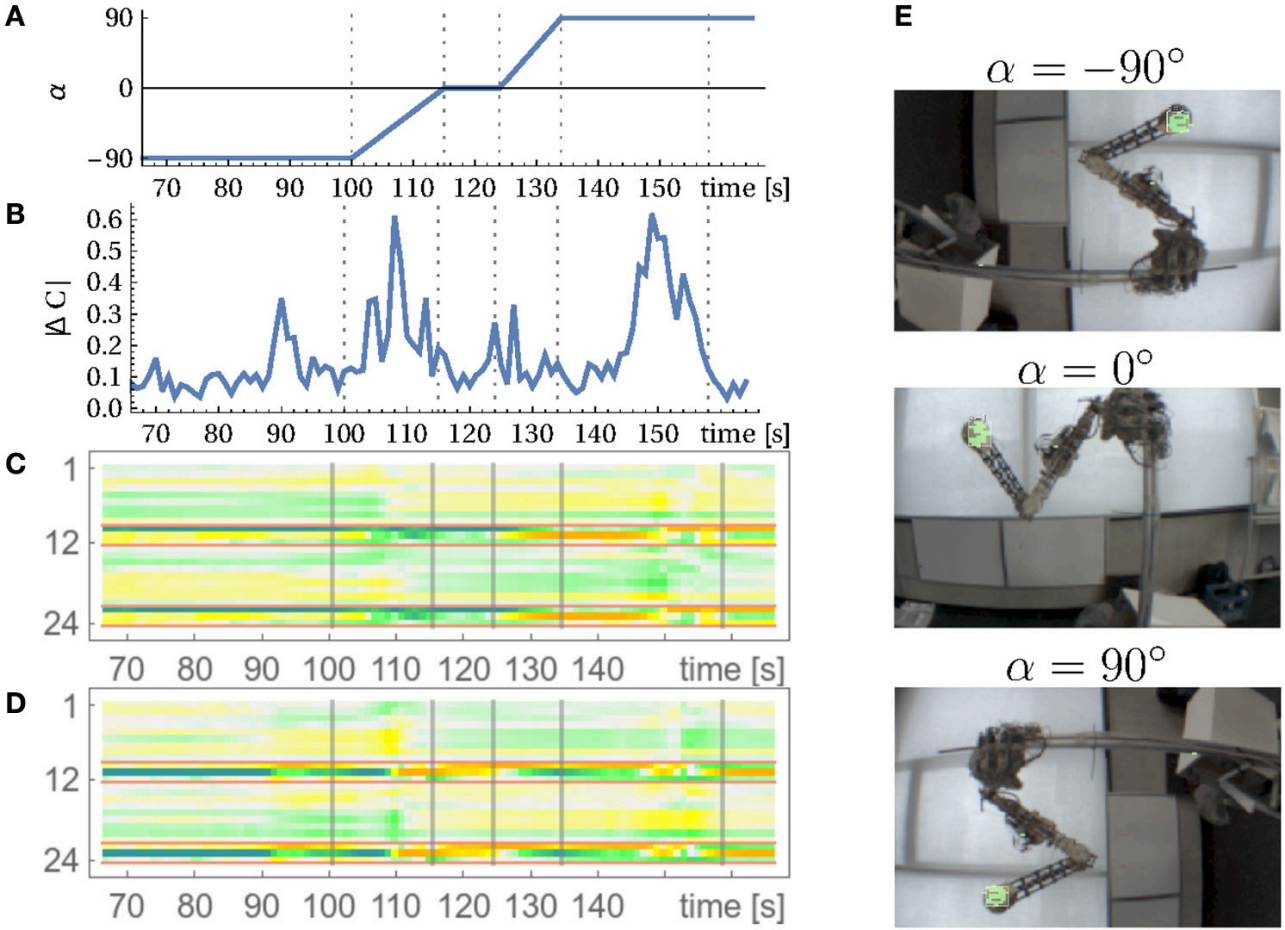
**Adaptation to disruptive changes in the vision sensors**. During the experiment the camera was rotated about its optical axis. **(A)** Camera angle in degrees and corresponding camera images in **(E)**. **(B)** Change of the controller matrix *C* over time (averaged over 1 s). **(C,D)** Evolution of the coupling of the 24 sensors to one muscle (3 and 8) over time (row 3 and 8 of *C* over time). Red-framed sensors are the vision sensors (and their time-delayed version). Vertical lines indicate times of camera rotation and the point of reentering a stable motion at 160 s. See corresponding Video 10 in Supplementary Material and the text for details.

In the previous experiments, the sensorimotor loop was closed in proprioceptive space alone, muscle lengths and tensions generating muscle feelings with the ensuing piloting effect, see Section 3.3. This section investigates the integration of additional sensors given by a camera reporting the spatial coordinates of a green colored object connected to the tip of the arm, called the *fist* in the following. The camera was positioned to observe the arm from the front, see Figure 3F, but other positions would also work. The *x*−*y* coordinates of the object are obtained from the green pixels' center of gravity, whereas the *z* coordinate is given by the size of the pixel cluster. These coordinates are scaled between -1 and +1 as all the other sensors. To better compete with the 9 proprioceptive sensors, the corresponding synaptic weights were multiplied by a factor of 3 (before normalization). No other measures were taken, in particular, all entries for the vision channels in the model matrix *M* were put to zero in accordance with the least biasing commitment described in Section 2.2. In the experiments, we observed that the robot engaged into all kinds of trajectories similar to those of the purely proprioceptive case, i.e., as if the camera were not present. However, a simple inspection of the *C* matrix reveals a strong involvement of the vision channels in the generation of the modes, see the red-framed rows in Figures 10C,D. The constitutive role of the camera can also be seen by the following experiment.

#### 3.8.1. Adaptation to sensor transformations—rotating the camera

In this setting we rotate the camera about its optical axis while the system is running and DEP learning is on, with a time scale of a few seconds. Initially the camera is rotated about its axis to -90 degrees, see Figure 10E. When a relatively stable motion occurs (limit cycle), the camera is slowly rotated to a normal orientation (0 degrees). During that process, the motion pattern of the arm changes until, after stopping the camera rotation, a new attractor behavior is reached. Together with Figure 10 this shows that the emerging patterns are generated with the camera closely integrated^5^. Eventually, upon rotating the camera further to +90 degrees, the motion of the arm even stops until, after about 15 s, a new consistent behavior emerges, see Video 10 in Supplementary Material and Figure 10. The experiment shows that DEP learning generates motion patterns with the camera tightly integrated, i.e., proprioceptive and vision channels are strongly mixed. We remark that readaptation and reorganization of behavior takes place on a time scale of a few seconds.

#### 3.8.2. Hand-eye coordination. emerging central pattern generator

**Figure 11:**
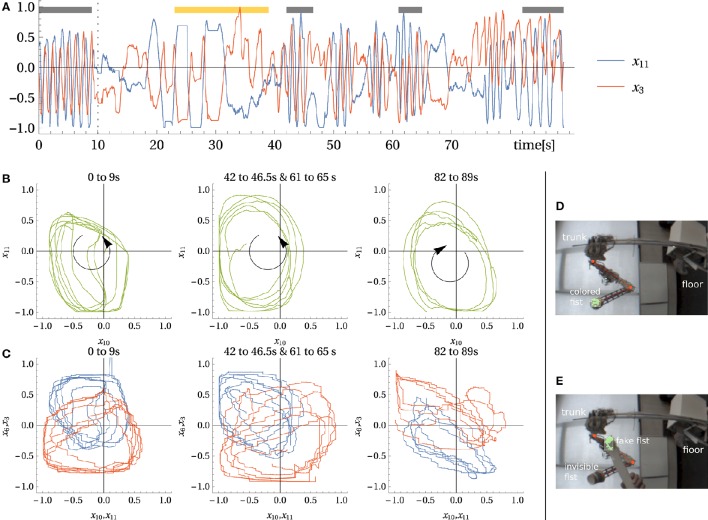
**Experiment with camera input. Hand-eye coordination and tracking. (A)** a proprioceptive sensor *x*_3_ and a vision sensor *x*_11_ (up-down direction) over time. The vertical line indicates when the fist was covered with a cap (see **E**). Black bars indicate time intervals used in **(B,C)**. The yellow bar indicates the cut out part of the corresponding Video 11 in Supplementary Material. **(B)** Trajectory in vision sensor space for different parts. Left: *original* movement (with normal camera sight **(D)**, middle: two *similar driven* behaviors, right: *inverted direction* movement. **(C)** The same trajectory relating vision to proprioception sensors *x*_11_ → *x*_3_ and *x*_10_ → *x*_6_. **(D,E)** camera picture for normal and dummy-fist case.

As discussed above, DEP learning potentially integrates all sensor channels, converging toward a fixed point in correlation space which corresponds to a periodic motion pattern in state space. This is seen from the parametric plots in Figure 11C, first row displaying a proprioceptive vs. one of the vision channels. Despite the strong perturbations in the complex physical setting, a distinct phase relation between vision and proprioception is seen. This is another corroboration of the integrative strength of DEP.

In a next experiment, we investigate the acquired sensorimotor mappings in more detail. During learning the camera delivers a periodic trajectory in a 3D space, correlated tightly with proprioception. What if we substitute the camera coordinates by those of a fake, or virtual, trajectory. In the experiment, we wait until the system, with the camera included, settled into a stable motion pattern. Then, we freeze the controller matrix *C* and cover the fist with a white cap making it invisible to the camera's green object detector so that the vision sensors are frozen. Now we use a dummy fist (green ball attached to a stick) to generate camera coordinates by hand, see Figures 11D,E for a normal and a dummy fist camera view, respectively.

As demonstrated in Video 11 (Supplementary Material), moving the dummy generates defined movements of the arm, although the arm would typically not follow the dummy if it is arbitrarily moved. However, if the dummy is moved along a similar path as the original movement, the arm is following the dummy, it can be even driven into trajectories with various velocities, and can be stopped deliberately, see Video 11 in Supplementary Material. In Figure 11A the time trace of one of the vision sensors and a proprioceptive sensor for the course of the experiment visualizes this behavior. By comparing the parametric plots in Figures 11B,C, first and second row we confirm the similarity between the original and the virtual camera trajectory. On the other hand, Figures 11B,C, third row shows that a different relation between the sensors occur if the dummy trajectory is in the opposite direction.

Another interesting point is that behaviors can not only be replayed and combined, as demonstrated in the wiping case, but also be driven by virtual trajectories with (moderately) varying shapes and velocities. This can be operationalized for deliberate control. For instance, a central pattern generator could be used to generate the virtual trajectory, giving the opportunity to systematically vary frequency and shape of the emerging behaviors. Furthermore, the emergence of hand-eye coordination and the possibility to deliberately control the arm using virtual trajectories could be of some interest for the development in infants during Piaget's first phase.

### 3.9. Perspectives for goal oriented behavior

Though this paper is devoted to robotic self-organization, let us have a remark on generating user chosen behaviors. The basic idea is the following: the classical control setting is a two level hierarchy where the goal driven controller is applied directly to the low level PID controller realizing the action execution. Here, we advocate for the inclusion of a third, intermediary level, meaning that the higher-level controller is realizing its goals by manipulating the above mentioned meta-system with its wealth of latent behaviors waiting to be excited. How this could be effectively done is still to be investigated. However, the potential success of this extended hierarchy of control is suggested by the experiments. In fact, if we are able to influence the meta-system by hand, why not by just superimposing additional motor signals on the self-regulated meta-system. The use of the approach is encouraged by the mentioned ability of the meta-system to uphold a resilient working regime even under extreme external perturbations, preventing, for instance, shoulder dislocations.

## 4. Discussion

This paper is seen as a further step toward a general theory and practical realization of self-organization (SO) for embodied AI. There are many facets to such a general idea worth to be investigated. In many cases, SO is considered as either self-exploration for scrutinizing the gross properties of the system (to be deliberately controlled afterwards), or (wishfully) used for the acquisition of behavior primitives. While this is often ticked-off as superfluous, to be replaced by well known methods like motor babbling, SO definitely has its realm if systems become larger. This has been demonstrated by a number of successful examples (Der and Martius, 2012, 2013, 2015; Der, 2016) attributing SO a much wider range of applicability. We claim that the results of this paper are a further step as they extend that range to composed systems consisting of the actual robot connected to a subsystem with an unknown internal dynamics. In the paper we ask how a robot may establish dynamical contact with a subsystem, eventually recognizing its identity, if there is no information or model of the subsystem's inner dynamics. Humans seem to have no problems there as they develop a feeling, by their muscle tensions, for the reactions of the subsystem. However, it is not clear what this subjective feeling is and how it is used for controlling the interacting system.

As a first insight offered by our DEP controlled robot, we note that the artificial system does not need any curiosity or other higher level concepts for producing the observed human like behaviors. Oriented at the similarity between our anthropomorphic robot and human behavior, we may question the ontological status of these higher level concepts also in humans. Furthermore, we could reveal a very subtle but dominating effect: by the mere feedback through the internal dynamics of the object, the robot is learning to answer with a very specific sensorimotor pattern to each of the objects. So, the robot discovers the identity of the attached object without knowing anything of its dynamical properties which may be very complex like the water in the bottle. This may be a further example how the robot can both model and substantiate concepts from cognitive science, here Gibson's object affordances. Furthermore, as we could uncover by the analytical tools developed in this work, the emergence of the combined mode and the eventual identification of the attached object—by establishing dynamical contact—is explained by a subtle mechanism which we call piloting.

Unfortunately, due to the high complexity of the system and the subtlety of the effect, a rigorous mathematical analysis is not possible so far. Nevertheless, using some concepts of dynamical system theory, we could establish tentative findings. By keeping the system at the border to instability we find a potentially infinite reservoir of (limit cycle) attractors “waiting” to be excited. Besides converging toward one of these attractors, the rich reservoir of further phenomena could possibly be related to concepts like attractor meta-dynamics (Gros, 2015; Sándor et al., 2015), the so called meta-transients (Negrello and Pasemann, 2008) and the mentioned self-induced attractor morphing. Altogether, these concepts may serve as a characteristic for self-organized behavior in the sensorimotor loop, possibly endowing even the edge of chaos concept with a new realm. There again, we emphasize that the outstanding sound mathematical analysis of these concepts can more reliably reveal their enormous potential for constructing and building such self-learning machines with their creative properties.

It is also important to note that “reading” the object's properties through the mere feedback from its internal dynamics is a direct consequence of those dynamical system properties. Considering the similarity with human behavior again, we may ask if humans also work in this dynamical regime at the border of instability and what the possible consequences are. It must be left to future work to reveal the thereby expected cross fertilization between robotics and cognitive science. Furthermore, the spontaneous identification of dynamical object affordances may be also of some interest for both robotics and embodied AI.

In short, we claim that experimental investigation with anthropomorphic, self-learning robots not only generates interesting behaviors in complex robotic systems. It may also help to better understand what subjective human feelings of physical interactions are, how they can be rooted in sensorimotor patterns, and how these concepts may feed back onto robotics. Hopefully, this knowledge may eventually help building machines that are as close to humans as possible.

Last but not least, let us briefly compare our results with the literature on SO in robotics. While this paper focuses on the SO of behavior for robots of a given morphology, much of the literature is devoted to SO for self-assembling and self-repairing (Murata and Kurokawa, 2012), and eventually self-replicating (Griffith et al., 2005) systems. Very influential for the topic is the paper Pfeifer et al. (2007) presenting the whole spectrum of bioinspired robotics. The central idea is that control is outsourced to the morphological and material properties, see also Hauser et al. (2012), Pfeifer and Gómez (2009), Paul (2004), Pfeifer and Bongard (2006), Pfeifer and Scheier (1999), and Pfeifer et al. (2012). This is in line with our work, as our controller is developing everything from the interplay with the physics of the system. However, to our knowledge previous work does not reach robots of such complexity as demonstrated here. Related to our work is the multiple attractor concept (Tani and Ito, 2003; Gros, 2015; Sándor et al., 2015), which was not yet applied to real robots. Another body of literature exists on SO in swarms (Bonabeau et al., 1997, 1999; Rubenstein et al., 2014; Blum and Groß, 2015) to get swarm intelligence (Engelbrecht, 2006; Nouyan et al., 2008), but there is no relation to our work which is devoted to the development of individual robots.

## Author contributions

RD and GM conceived and conducted the experiments. GM analyzed the data. RD and GM wrote the paper.

### Conflict of interest statement

The authors declare that the research was conducted in the absence of any commercial or financial relationships that could be construed as a potential conflict of interest.

## Footnotes

^1^This needs a regularization, i.e., in the experiments we use κ_*i*_ = κ/(||*C*_*i*_|| + λ) with λ > 0 is very small.

^2^This effect involves the normalization factors and fades away once the regularization comes into play. After that, the system tries to move to the global attractor ẋ = *x* = 0.

^3^Note that later in the experiment, the string of the pendulum was shortened such that a different sensorimotor coordination emerges.

^4^This is true in particular for the other complex eigenvalue with roughly half the value, apparently belonging to a subharmonics but this still needs some more analysis.

^5^During a periodic motion pattern the controller matrix *C* stays relatively constant, i.e., a fixed point in correlation space is reached.
