# Supplementary material for: Self-Organized Behavior Generation for Musculoskeletal Robots
Source: Front Neurorobot. 2017 Mar 16;11:8. doi: 10.3389/fnbot.2017.00008 (PMC5352682; doi:10.3389/fnbot.2017.00008)
Supplement: Supplementary file 1 [file Appendix.pdf]

# 5 APPENDIX

In this section we derive the controller matrix $C$, discuss the role of the sensor to motor mapping matrix $M$, and give the update rule for $C$.

## 5.1 The controller

In order to derive Eq. (5), we need to predict $\dot{x}'_t = \dot{x}_{t+1}$ on the basis of the previous sensor values. Our idea is based on an operator [6]

$$L_t = \dot{x}'_t \hat{x}_t^\top \tag{10}$$

where $\hat{x} = \|\dot{x}\|^{-2}\dot{x}$, which describes the transition $t \to t+1$ as

$$\dot{x}'_t = L_t \dot{x}_t \tag{11}$$

As to notation, we remark that with any two column vectors $a$ and $b$, $a^\top b$ is the scalar product and $S = ab^\top$ is a matrix with elements $S_{ij} = a_i b_j$. In particular, $\dot{x}^\top \dot{x} = \|\dot{x}\|^2$ and $\hat{x}^\top \dot{x} = 1$, which corroborates Eq. (11).

Of course, $L_t$ cannot be used directly as it involves the future. The idea is to generalize that expression in a way which is consistent for trajectories with some time coherence. Here, we replace $L$ with its moving average, defined as

$$\bar{L}_t = \frac{1}{Z}\sum_{s=1}^{t-1} \tau^{t-1-s} L_s = \sum_{s=1}^{t-1} p_s \dot{x}_{s+1} \dot{x}_s^\top \tag{12}$$

where $\tau < 1$ defines the time scale for the extension of the past and $Z = \sum_{s=1}^{t-1} \tau^{t-1-s}$ and $p_s = \frac{1}{Z}\tau^{t-1-s}\|\dot{x}_s\|^{-2}$ are normalization and weighting factors, respectively, and we used $\dot{x}'_s = \dot{x}_{s+1}$ in the last step. Writing $x_i(s)$ for the i-th component of the vector $x_s$ and similarly for $\bar{L}_t$, the matrix elements of $\bar{L}_t$ are

$$\bar{L}_{ij}(t) = \sum_{s=1}^{t-1} p_s \dot{x}_i(s+1)\dot{x}_j(s) \tag{13}$$

and in shorthand $\bar{L}_{ij}(t) = \langle \dot{x}'_i \dot{x}_j \rangle_P^{t-1}$, where $p_s = \frac{1}{Z}\tau^{-s}\|\dot{x}_{s-1}\|^{-2}$ are weighting factors. Note that $\bar{L}$ does not any longer involve the future as it is shifted by one step in time and that the time coherence can be controlled by the decay term $\tau$. Remembering that $\dot{x}_t = x_t - x_{t-1}$ we see that $\bar{L}$ is given by the history of the sensor values in a definite way.

We may now use Eq. (13) as dynamical operator so that

$$\dot{x}'_t = \bar{L}_t \dot{x}_t \tag{14}$$

generates a time series: given the history $\dot{x}_t, \dot{x}_{t-1}, \ldots$, we get the future evolution of $\dot{x}$ by iterating Eq. (14). Note that the history until $t$ defines the future of the time series in a deterministic way so that there are as many time series as there are different histories. Because of this generality, we may call Eq. (14) with Eq. (13) a template defining a certain class of time series. Note there are no parameters involved, apart from the time scale of the history as set by $\tau$.

Using Eq. (14) we may express the future state $\dot{x}'$ in terms of its history. Taking the time derivative of Eq. (4) yields $\dot{y} = M\dot{x}'$, assuming $M$ is constant. Putting $y = g(z)$ where $g$ is the squashing function, we

[6] Actually, $L$ is simply a matrix but we call it an operator for emphasizing the dependence on the states it is operating on.

obtain for the internal control variable $z$, ignoring the nonlinearity of the squashing function

$$\dot{z}_t \approx C_t \dot{x}_t \tag{15}$$

where

$$C_t = M_t \bar{L}_t \tag{16}$$

In a final step we have to relate $\dot{z}$ to $z$ in a way that is consistent with the postulated slowly varying nature of $\bar{L}$. In this paper we use the most simple postulate, i. e. omit the residual time dependence of C altogether. Using the simple relation $x_t = \sum_{s=1}^{t} \dot{x}_s + x_0$ and analogously for $z_t$, we define our controller as

$$y_t = g(C_t(x_t - x_0) + z_0)\,, \tag{17}$$

where $z_0$ is an overall bias that can be set arbitrarily, each $z_0$ leading to a different control strategy. This versatility is a direct result of working with the $\dot{x}$. $z_0$ may also be adapted following a heuristics like avoiding a saturation regions of the squashing function or coping with an overall bias of the system. $x_0$ plays the role of a sensor bias. In this paper, $x_0 = 0$ as our systems are centered around $x = 0$. Otherwise it can be adapted[7] similarly to $z_0$. In the experiments we used the controller

$$y_t = g(C_t x_t) \tag{18}$$

throughout, where $g_i(z) = \tanh(z_i)$.

For a brief discussion we assume that $x_t$ follows a harmonic oscillation with period $T$ and ask whether this is consistent with the explicit form of $\bar{L}$ as given by Eq. (12). Linearizing Eq. (18) so that $x' = My \approx MCx = \bar{L}x$, we have to consider the role of $\bar{L}$ as applied to $x$. Considering Eq. (12) together with $\dot{x}_t^\top x_t = \dot{x}_{t-T/2}^\top x_t = 0$ and $\dot{x}_{t-T/4} \propto x_t$, we may argue[8] that $\bar{L}x_t \approx \dot{x}'_{t-T/4} \propto x'_t$. This crude argument which repeats for each half-period shows that the controller is consistent with the stipulated sensor dynamics, provided the mapping $M$ can translate this into appropriate motor commands. Note also that the time smoothing in $\bar{L}$ does not mean a time smoothed dynamics as the above argument remains valid for any frequency (below the Nyquist frequency for the given update rate).

## 5.2 The role of $M$

Central to the approach is the template dynamics Eq. (14). In general, any real dynamics will not fit into that template, i. e. the true dynamics in sensor space generated by the robot can be written as

$$\dot{x}'_t = \bar{L}_t \dot{x}_t + \xi_t \tag{19}$$

where $\bar{L}$ is given by Eq. (13) in terms of the real trajectory as generated by Eq. (19). Let us now hypothetically assume that $A$ represents exactly the sensor response of the arm to the motor actions, i. e. assume $\dot{x}' = A\dot{y}$ with $A = M^{-1}$ and $\xi = 0$. Then, the pair of Eqs. [3, 4] degenerates into a triviality so that the system can realize any trajectory. So, the actual point of interest is the mismatch, represented by $\xi$, between true behavior and the template. As a rule of thumb we postulate that the controller is able to realize a trajectory the better the smaller $\xi$. The mismatch $\xi$ directly reflects the physical reactions of the meta-system to the motor actions. In this light, trajectories are more stable for more systematic reactions,

[7] Actually, $x_0$ is the state at time $t = 0$. However, even by very small perturbations or the residual time dependence of $C$, the memory of the initial state is soon lost so that we are free to choose the sensor bias.

[8] Note that the norm of the rotating vector is constant and assume $\tau \approx 1$, i. e. the weighting factors $p_s$ are independent of $s$.

involving the degrees of freedom of the physical system in a coherent manner. This is what we may observe in the experiments.

The standard version is that $M$ is to reflect just the most basic causal relations between sensor and motor signals. $M$ can be learned in simple off-line motor babbling scenarios, see Der and Martius (2015) for examples. In this paper, we used the relation between motor encoder and tendon length which is a one-to-one mapping, hence $M$ is the unit matrix (identity operator). This choice also underlines the difference between the matrix $M$ and the usual understanding of an internal inverse model: while the latter is to reflect the mapping from sensors to motors as precisely as possible, $M$ determines $\xi$—the mismatch between template and true dynamics which determines both the self-exploration rate and the regularity of the generated motion patterns as a (very complex) function of the character parameters ($\tau$, $\kappa$). More details on this search and converge paradigm may be found in Der and Martius (2015).

## 5.3 Eliciting periodicity

To make the system more attractive for motion patterns we introduce additional sensor values which are delayed copies of the original sensors. The time lag is given by the meta-parameter $d$. In the following we assume the system is already in a periodic motion with period $T = 2d$. Let us denote the delay-sensor values as $x_{t-T/2}$ together with a second controller matrix $C_{t|-T/2}$ and define the vector of the motor commands as

$$y_t = g(C_t x_t + C_{t|-T/2} x_{t-T/2}) \tag{20}$$

where, before normalization,

$$C_{t|-T/2} = M_{t|-T/2} \langle \dot{x}'_i \dot{x}_j \rangle_P^{t-T/2-1} \tag{21}$$

and $M_{t|-T/2}$ transforms motor values at time $t$ into sensor values at time $t - T/2$. Note, that $M_{t|-T/2}$ can be different than $M_t$. If the system is in a periodic regime we have $x_t = -x_{t-T/2}$ and thus also $\langle \dot{x}'_i \dot{x}_j \rangle_P^{t-1} = \langle \dot{x}'_i \dot{x}_j \rangle_P^{t-T/2-1}$. Choosing $M_{t|-T/2} = -M_t$, we find $C_{t|-T/2} = -C_t$ so that, before the normalization, the argument of the squashing function ($\tanh$) is given by $C_t x_t + C_{t|-T/2} x_{t-T/2} = 2C_t x_t$. Hence, there is a constructive interference over the half period so that periodic patterns are getting favored for self-amplification. This argument holds true also for any multiple of the fundamental period $T$.

## 5.4 Some technical details

In the practical applications done so far, we identified a number of tweaks for coping with peculiarities of the approach. One is the choice of the time lag between $x$ and $x'$ which was $x'_t = x_{t+1}$ above. However, there is no hindrance to introduce a certain lag $\theta$ so that $x'_t = x_{t+\theta}$. This is helpful in order to adapt the system to the actual update rate and in particular for enhancing the chance for periodic patterns. It also influences to some extent the frequency of such patterns.

Another point concerns the regularization of the normalization factors which have different effects for either the normalization of $C$ or that of $\bar{L}$ which was introduced with the $p_s$ factors Eq. (13). As the former normalization acts on $C$ directly, its effect is delayed on the time scale given by $\tau$. The normalization of $\dot{x}$ on its hand also needs some regularization, i. e. we have to replace the factor $||\dot{x}||^{-2}$ as

$$||\dot{x}||^{-2} \rightarrow \frac{1}{||\dot{x}||^2 + r} \tag{22}$$

where $r$ may run in principle from $10^{-1}$ down to a minimal value determined by the discretization of the sensor values. However, very small $\dot{x}$ are enlarged up to a factor $r^{-1}$ and dominate in this way the definition of $C$. This is not helpful as in most cases the very small velocities arise from e. g. sensor noise, which tends to destroy the already reached self-amplification of latent behavior. In practice, it is therefore helpful to keep the regularization effect in bounds. A values of $r = 10^{-3}$ seems to be a good choice.
