# Supplementary material for: Self-Organized Behavior Generation for Musculoskeletal Robots
Source: Front Neurorobot. 2017 Mar 16;11:8. doi: 10.3389/fnbot.2017.00008 (PMC5352682; doi:10.3389/fnbot.2017.00008)
Supplement: Supplementary file 2 [file Videoswithcaptions.PDF]

Supplementary material for:
Self-organized behavior generation for musculoskeletal robots

Ralf Der and Georg Martius

December 2, 2016

1 Overview

The videos can be watched at http://playfulmachines.com/MyoArm-1

Handshake	Human robot interaction by manually imposing a periodic movement	Video 1
Arm with pendulum	Suspending a weight from the tip of the arm: self-excitation of a circular pendulum mode	Video 2
Pendulum responses	Motors are stopped. Recording spring forces of swinging suspended bottle	Video 3
Shaking horizontally	A half filled bottle is horizontally attached to the tip of the arm: shaking of the bottle mainly along its axis	Video 4
Shaking vertical	Vertical attachment, half filled: shaking direction mainly along the (now vertical) axis	Video 5
How to rotate a wheel	Arm attached frontally to a revolvable bar/wheel.	Video 6
Rotating wheel II	Parallel wheel – arm arrangement	Video 7
Wiping table	Arm with brush starts to wipe a table	Video 8
Wiping table modes	Different wiping patterns from reloaded controllers	Video 9
Sensor disruptions	With visual input for hand. Camera is turned during behavior. Fast reorganization	Video 10
Hand-eye coordination	Coordination develops, such that arm follows a dummy hand	Video 11

2 Videos

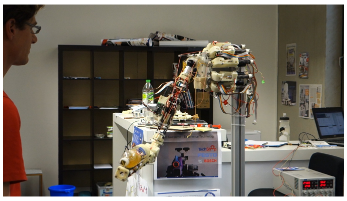

Video 1: Handshake: Human-robot interaction by manually imposing a periodic movement. A longer version can be found here. See http://playfulmachines.com/MyoArm-1.

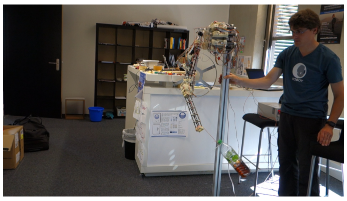

Video 2: Bottle swing: Excitation of a circular pendulum mode. The suspended bottle, once exited to swing a little bit, excerts forces onto the arm, which are incoorporated into the controller throught the plasticity rule. This leads eventually to a coherent swinging motion. See http://playfulmachines.com/MyoArm-1.

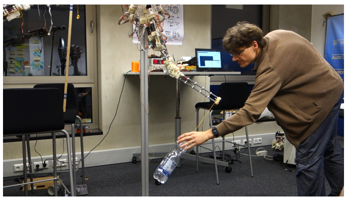

Video 3: Pendulum responses: Motors are stopped. Recorded are the spring forces of a swinging suspended weight. See the figure in the paper for the resulting sensor readings. See http://playfulmachines.com/MyoArm-1.

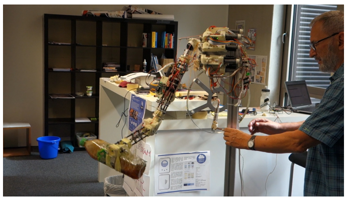

Video 4: Shaking horizontally: A helf filled bottle is horizontally attached to the tip of the arm. The main shaking direction is horizontal. Stronge response to dynamics inside the bottle. See http://playfulmachines.com/MyoArm-1.

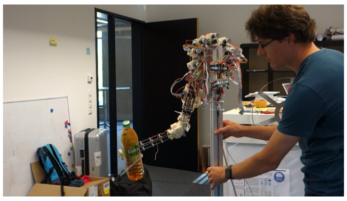

Video 5: Shaking vertically: A half filled bottle is vertically attached to the tip of the arm. Emergent shaking behavior of the bottle mainly along its axis. See http://playfulmachines.com/MyoArm-1.

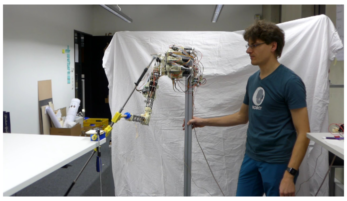

Video 6: How to rotate a wheel: Arm is attached frontally to a revolvable bar/wheel. In the beginning, the arm is very loosely attached to the crank so that there is no definite force transfer. After improving the connection and some kick by the experimenter the arm rotates the wheel. It can then also quickly learn to rotate the other direction. See http://playfulmachines.com/MyoArm-1.

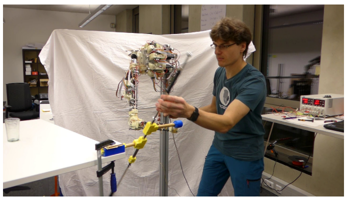

Video 7: Rotating wheel II: Parallel wheel – arm arrangement. The arm is self-learning to rotate the wheel and also quickly learns to rotate in the opposite direction. See http://playfulmachines.com/MyoArm-1.

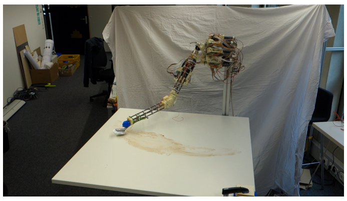

Video 8: Wiping table: Arm with brush starts to wipe a table. The table plane and the manual guidance makes the to arm to wipe the table. Later in the video a different wiping pattern is generated. See http://playfulmachines.com/MyoArm-1.

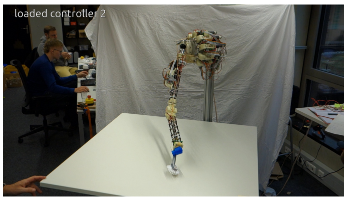

Video 9: Wiping table modes: Different wiping patterns from reloaded controllers. Controller that where saved during a previous run where reloaded one after another. One observes smooth transitions between the different wiping modes, an example of the attractor morphing discussed in the paper. Occasionally, transitions may take some time. See http://playfulmachines.com/MyoArm-1.

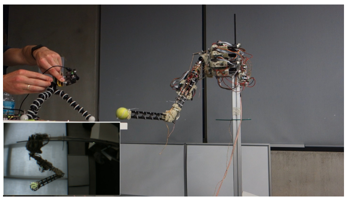

Video 10: Including vision I: The camera coordinates for the green fist are used as additional sensor values. With fixed camera, cyclic arm motions emerge. When the camera is slowly rotated, these patterns are coherently morphing into a new pattern after the camera was being stopped. See http://playfulmachines.com/MyoArm-1.

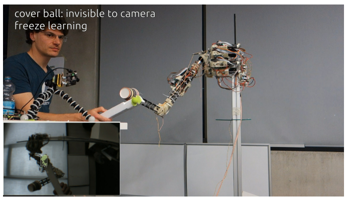

Video 11: Including vision II: Emerging Fist-eye coordination: After a stable motion pattern has developed, the fist is capped but the camera now sees the dummy fist. Observe how the arm follows the dummy fist in a deliberate but irregular manner. However, the dummy can guide the arm in a coherent motion if it runs along the original trajectory. See http://playfulmachines.com/MyoArm-1.
